# *De novo *sequencing of circulating miRNAs identifies novel markers predicting clinical outcome of locally advanced breast cancer

**DOI:** 10.1186/1479-5876-10-42

**Published:** 2012-03-08

**Authors:** Xiwei Wu, George Somlo, Yang Yu, Melanie R Palomares, Arthur Xuejun Li, Weiying Zhou, Amy Chow, Yun Yen, John J Rossi, Harry Gao, Jinhui Wang, Yate-Ching Yuan, Paul Frankel, Sierra Li, Kimlin Tam Ashing-Giwa, Guihua Sun, Yafan Wang, Robin Smith, Kim Robinson, Xiubao Ren, Shizhen Emily Wang

**Affiliations:** 1 Department of Cancer Biology, City of Hope Beckman Research Institute, 1500 Duarte Road, Duarte, 91010, USA; 2 Department of Molecular Medicine, City of Hope Beckman Research Institute, 1500 Duarte Road, Duarte, 91010, USA; 3 Department of Medical Oncology, City of Hope Medical Center, 1500 Duarte Road, Duarte, 91010, USA; 4 Department of Population Sciences, City of Hope Beckman Research Institute, 1500 Duarte Road, Duarte, 91010, USA; 5 Department of Molecular and Cellular Biology, City of Hope Beckman Research Institute, 1500 Duarte Road, Duarte, 91010, USA; 6 Department of Biostatistics, City of Hope Beckman Research Institute, City of Hope Comprehensive Cancer Center, 1500 Duarte Road, Duarte, 91010, USA; 7 Department of Immunology & Biotherapy, Tianjin Cancer Institute & Hospital, Tianjin Medical University, Tianjin, 300060, China

**Keywords:** Breast cancer, miRNA, Biomarker, Neoadjuvant chemotherapy, Metastasis

## Abstract

**Background:**

MicroRNAs (miRNAs) have been recently detected in the circulation of cancer patients, where they are associated with clinical parameters. Discovery profiling of circulating small RNAs has not been reported in breast cancer (BC), and was carried out in this study to identify blood-based small RNA markers of BC clinical outcome.

**Methods:**

The pre-treatment sera of 42 stage II-III locally advanced and inflammatory BC patients who received neoadjuvant chemotherapy (NCT) followed by surgical tumor resection were analyzed for marker identification by deep sequencing all circulating small RNAs. An independent validation cohort of 26 stage II-III BC patients was used to assess the power of identified miRNA markers.

**Results:**

More than 800 miRNA species were detected in the circulation, and observed patterns showed association with histopathological profiles of BC. Groups of circulating miRNAs differentially associated with ER/PR/HER2 status and inflammatory BC were identified. The relative levels of selected miRNAs measured by PCR showed consistency with their abundance determined by deep sequencing. Two circulating miRNAs, miR-375 and miR-122, exhibited strong correlations with clinical outcomes, including NCT response and relapse with metastatic disease. In the validation cohort, higher levels of circulating miR-122 specifically predicted metastatic recurrence in stage II-III BC patients.

**Conclusions:**

Our study indicates that certain miRNAs can serve as potential blood-based biomarkers for NCT response, and that miR-122 prevalence in the circulation predicts BC metastasis in early-stage patients. These results may allow optimized chemotherapy treatments and preventive anti-metastasis interventions in future clinical applications.

## Background

Breast cancer (BC) is the most common cancer among females and a leading cause of cancer death worldwide [1]. Current clinical decision-making for BC treatment relies on tumor characteristics and therapeutic targets including the estrogen receptor (ER), progesterone receptor (PR), and human epidermal growth factor receptor 2 (HER2). Chemotherapy is given in the neoadjuvant and adjuvant settings to patients with locally advanced/high-risk primary BC as treatment for metastatic BC leading to life-threatening parenchymal disease, or to treat endocrine resistant (ER/PR-negative) metastatic BC [2,3]. Neoadjuvant chemotherapy (NCT) is increasingly being used for initial treatment of locally advanced and inflammatory BCs. Pathologic complete response (pCR), defined as the preoperative eradication of tumors from the breast and axillary lymph nodes [4], is associated with optimal clinical outcome, including improved disease-free and overall survival [5,6]. However, conventional NCT regimens result in pCR in only 10-30% of treated BC patients [6]. In patients with residual invasive disease after NCT, a substantial proportion experience disease progression to metastatic stage within a few years after surgical resection. Patients with both *de novo *and recurrent metastatic BC face poor prognosis, with a median survival of 1-2 years and a 5-year survival rate < 20% [7-9]. Development of early diagnostic markers capable of predicting a patient's response to therapy and recurrence with metastatic BC is therefore critical to advancing more effective, personalized treatment. In this study, we explored the use of circulating miRNAs as blood-based, minimally invasive biomarkers for NCT response and disease relapse in locally advanced and inflammatory BC patients.

MiRNAs are naturally-occurring, non-coding small RNA molecules of 21-24 nucleotides (nts) that form partially complementary base pairs within the 3' untranslated regions of protein-encoding mRNAs, resulting in mRNA destabilization and/or translational inhibition [10]. To date, approximately 1000 miRNAs have been identified in humans. Compared to the large number of mRNA genes (~30, 000 mRNAs per cell), this relatively small number of miRNAs allows for large-scale evaluation for individualized diagnosis and treatment with higher efficiency and lower cost. Increasingly, reports demonstrate applications of miRNAs as tissue-based markers for the classification and prognosis of several human cancers, including BC [11-14]. A number of miRNAs have been found differentially expressed between BC and normal tissues, with significant up- (e.g., miR-21 and miR-155) or down-regulation (e.g., miR-10b and miR-145) in cancerous tissues [12,15,16]. Expression of certain BC-related miRNAs has been correlated with specific biopathologic features, such as ER and PR expression, tumor stage, vascular invasion, and proliferation index [12,14-18].

MiRNAs are stably present in whole blood, serum, and plasma [19,20]. Therefore, assessment of circulating miRNA profiles from cancer patients allowing for correlations between tumor traits (e.g., treatment response and metastatic potential) and cancer-released miRNAs are of great clinical interest. Using PCR assessments of selected miRNAs that are reportedly dysregulated in BC, several recent studies indicate associations of different circulating miRNAs with primary BC, metastatic disease, and ER status [21-25]. Microarray-based profiling has also been carried out in pilot studies, in which certain circulating miRNAs exhibit promising role as BC diagnostic markers [26,27]. Results from these previous studies, however, share limited consistency, possibly due to the different sensitivity and specificity of the detection approaches, as well as different patient numbers and compositions in the study cohorts.

Because some miRNAs may exclusively exist in the circulation with low or undetectable levels in cancer cells, powerful discovery approaches, such as deep sequencing analysis, may be more likely to identify diagnostic miRNA markers in the blood. Accordingly, we set out to explore the potential of deep sequencing in the current study to comprehensively analyze miRNAs that can serve as blood-based markers for NCT response and relapse with metastasis. As the first exploration to profile circulating miRNAs in BC patients using a *de novo *sequencing strategy, our study revealed global patterns of circulating miRNAs, and led to the identification of miRNA markers that can predict clinical outcome in primary stage II- III BC.

## Methods

### Study cohort and validation cohort

The clinical and histopathological factors of the study (training) and validation (testing) cohorts are summarized in Additional file 1: Table S1. Patients at City of Hope Medical Center (COH) had given informed consent before blood sera were collected under Institutional Review Board (IRB)-approved protocols, aliquoted and stored at -80°C until use.

Serum specimens of the training cohort were obtained as part of an NCT clinical trial conducted at COH. Forty-two female stage II-III BC patients deemed appropriate for NCT at the time of diagnosis were collected between December 2005 and April 2009. Upon diagnosis, all patients received conventional chemotherapy lasting 4-6 months followed by surgical resection of the tumor. Among the 42 patients, 7 received docetaxel/doxorubicin/cyclophosphamide (TAC) treatment regimen (group A), and 12 received doxorubicin/cyclophosphamide (AC) treatment followed by carboplatin and nab-paclitaxel (group B). The other 23 patients had HER2^+ ^BC, and were given the same regimen as for group B but with addition of trastuzumab (group C). One of the HER2^+ ^patients was likely stage IV based on presence of pleural effusion at diagnosis, which later was documented to be malignant. Biopsies of the primary tumor were analyzed for pathological classification. Upon completion of NCT, patients with Symmans residual cancer burden (RCB) score [4] of 0 were defined as pathologic complete response (pCR), whereas those with RCB score of ≥ 1 were defined as non-pCR. In the training cohort, 11 patients from all 3 treatment groups relapsed with metastatic disease within 1.5 years after serum collection, whereas the other 31 patients have not had disease progression to date during 2-5 years of follow-up. Patients with or without metastatic progression exhibit balanced age, tumor subtype, sample collection time and treatment regimen.

For the testing cohort, patients were selected from the COH Circulating Breast Cancer Tumor Marker Registry, an observational cohort study that recruits women with a variety of breast tumor histologies at the time of diagnosis, collects pretreatment biospecimens, and follows them throughout their clinical course, recording patient and tumor characteristics, treatments delivered, and clinical outcomes. Patients who had stage II-III BC at the time of registration who developed systemic recurrence while on study were defined as cases (*N *= 9). Eight of these had sufficient serum RNA for inclusion in the study. Controls were matched for 2:1 (*N *= 18) from the remaining stage II-III BC patients who had not developed systemic relapse on study and who had been followed at least as long as the case. All controls had sufficient serum RNA for the study. Other matching characteristics included age at diagnosis, hormone receptor and HER2 expression, and lymph node involvement. These patients were recruited onto the parent study between July 2006 and May 2010, a similar era to the training cohort; however, as an observational cohort, their systemic therapy regimen was determined by their primary oncologist. Half of the patients received a similar regimen to the training cohort, including a taxane with either doxorubicin, cyclophosphamide, and/or carboplatin, plus trastuzamab if their tumor was HER2^+^. The remaining individuals received a hormonal regimen for ER^+^HER2^- ^BC. All therapies were delivered in the adjuvant rather than neoadjuvant setting; therefore, metastatic relapse was the only measurable clinical outcome in this group. Mean follow-up for the testing cohort was 5.8 years.

### RNA purification from serum

TRIZOL LS reagent (Invitrogen) was used to extract total RNA from ~1.5 ml of serum, as describdi in the manufacturer's protocol. RNA pellet was dissolved in 10 μl of RNase-free water, and subjected to deep sequencing and qRT-PCR as described below.

### Solexa deep sequencing for small RNAs

Each serum sample was independently subjected to library preparation and deep sequencing. All small RNAs of 15-52 nts were selected and sequenced using the Solexa system, following the manufacturer's protocol (Illumina Inc., San Diego, CA). Library preparation, as well as cluster generation and deep sequencing, was performed according to the 5' ligation-dependent (5' monophosphate-dependent) manufacturer's protocol (Digital Gene Expression for small RNA; Illumina). For each sample, 5 μl of total RNA extracted from serum was used for small RNA library preparation. Small RNAs were size-selected between 17 and 52 nt according to the single-stranded DNA marker in the small RNA sequencing kit (Illumina). The library was quantified using picoGreen and qPCR. Sequencing was performed on a Genome Analyzer IIx (Illumina), and image processing and base calling were conducted using Illumina's pipeline.

### MiRNA-directed and genome-wide interrogation of identified sequences

Sequenced reads from Solexa were first mapped onto human genome version hg18 using novoalign software and the expression level of mature miRNAs in the miRBase human miRNA database v15 was summarized as described before [28]. Normalization and identification of differentially expressed miRNAs between groups were carried out using Bioconductor package "edgeR" [29].

### Reverse transcription (RT) and real time quantitative PCR (qPCR)

For qRT-PCR assays, 5 μl of total RNA extracted from serum was used as input into the RT reaction. RT was carried out using the miScript Reverse Transcription Kit (Qiagen) according to the manufacturer's protocol. For qPCR amplification, RT product was combined with PCR assay reagents containing miScript Primer Assay, Universal Primer, and SYBR Green PCR Master Mix (Qiagen). Real-time qPCR was carried out on a BioRad iQ5 thermocycler.

### Statistical analyses

Sequence data analysis and statistical comparisons were carried out using Bioconductor packages and an in-house developed analysis pipeline using R statistical environment. After mapping the deep sequencing data onto the human genome and counting the reads for the mature miRNAs in the miRBase database, raw miRNA expression data were quantile normalized and log2 transformed with offset of 1. Predictive miRNA classifiers for clinical outcome (the miR-375/miR-122 two-gene signature, and each gene individually) in the NCT training cohort was evaluated using univariate logistic regression and leave-one-out cross-validation. Briefly, one sample was left out as the test sample, and the remaining 31 samples served as the training set and used to train a univariate logistic regression model using the two-gene signature, which was then used to predict the status of the left out sample. If the predicted probability from the logistic regression model is more than a cutoff determined by minimizing the prediction error rate of training samples, the predicted status of that sample would be assigned as "relapsed", or "non-relapsed" otherwise. The predicted classification was then compared to the observed relapse status using 2-by-2 tables, from which sensitivity and specificity were calculated. This procedure was then repeated for each of the two single gene markers. To evaluate the performance of the two-gene signature in predicting the independent validation cohort, the entire NCT cohort (32 samples with RT-PCR data) was used as a training set. Odds ratio and 95% confidence internal were calculated using univariate unconditional logistic regression to determine if the histopathological parameters and circulating miRNAs were associated with NCT response.

## Results

### Study design, deep sequencing and annotation strategy

To comprehensively profile all small RNA species in the circulation, we isolated total RNA from ~1.5 ml serum collected from BC patients at initial diagnosis (prior to NCT), and selected small RNAs of 15-52 nts for library preparation and deep sequencing. The profiling study involved 42 stage II-III BC patients who participated in a clinical trial comparing docetaxel, doxorubicin, cyclophosphamide versus doxorubicin and cyclophosphamide, followed by nab-paclitaxel and carboplatin. All patients signed voluntary informed, IRB-approved consent forms, and were treated with NCT at the City of Hope Medical Center (ClinicalTrials.gov; NCT00295893). Among them, 11 relapsed with metastatic disease progression to stage IV during the follow-up, and the other 31 non-progressive control cases had matched ages, tumor subtypes and follow-up time, thus comprising the study cohort for metastatic relapse. The 23 HER2^+ ^patients received the trastuzumab-containing NCT regimen, upon which comparable numbers of pCR (12 cases; 52%) and non-pCR (11 cases; 48%) were observed. Correlation of miRNAs to NCT response and metastatic relapse was examined (see Methods and Additional file 1: Table S1 for details).

Total sequence reads obtained from each serum sample were first aligned to human genome database NCBI36/hg18 to overview the composition of circulating small RNAs and to identify known RNA species. Among aligned reads (~7-12 million) in all sequenced samples, miRNAs represented ~50% of all read counts. Other detected small RNA populations included tRNA (~28%), small cytoplasmic RNA (scRNA; ~8.8%), rRNA (~4.4%), small nuclear RNA (snRNA; ~0.6%), and small nucleolar RNA (snoRNA; ~0.4%), etc. (Additional file 1: Figure S1).

### Identification of circulating miRNAs associated with clinical parameters

**Figure 1:**
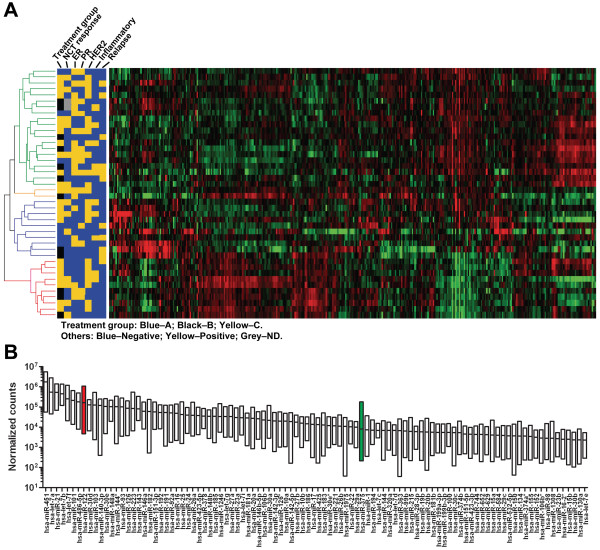
**MiRNAs detected in circulation**. **A**. Unsupervised hierarchical clustering over all detected miRNAs. Colors in the heatmap represent a sample's higher (red) or lower (green) level in relation to the mean of all samples. The status of each clinical parameter was represented by colored boxes as indicated in the figure. **B**. The top 100 miRNAs with the highest quantities in circulation. For each miRNA shown, the range of normalized counts was indicated by a column, and the mean value was indicated by a line. Arrows indicate the positions of miR-122 and miR-375 in the order of abundance.

MiRNAs were assessed and were deemed detectable if seen in at least 2 patients. This resulted in detection of more than 800 miRNAs, including 373 miRNAs with sequence counts of > 50 in at least 10% of the samples (Additional file 2: Table S2). Unsupervised hierarchical clustering of these miRNAs detected in the circulation separated ER^+ ^from ER^-^cases (Figure 1A). No perfect separation was observed between relapsed and non-relapsed cases at the global level. The top 100 circulating miRNAs with the highest average counts among all tested patients are indicated in Figure 1B (see Additional file 2: Table S2 for a full list of all detected miRNAs). These include several miRNAs that had been measured by PCR in previous studies, such as miR-21, let-7a, miR-155, and miR-10b, as well as miR-375 and miR-122, the two genes we subsequently identified as outcome-associated genes.

We next analyzed the association of each miRNA with a given clinical parameter, i.e., status of relapse, NCT response, ER/PR/HER2 expression or inflammatory disease. Multivariate comparison, taking into account the different NCT regimens the patients received, led to the identification of two miRNAs, miR-375 and miR-122, that were differentially expressed between patients who later developed metastatic relapse and those who did not, with *P *< 0.005 and false discovery rate (FDR) < 0.1 (Table 1). Because only 2 HER2^- ^patients in the whole 42-patient cohort had pCR to NCT, the comparison on treatment response was only carried out among HER2^+ ^patients, all of whom received the same trastuzumab-containing NCT regimen. We identified 7 miRNAs that were associated with NCT response (pCR vs. non-pCR) in HER2^+ ^patients with FDR < 0.1 (Table 1). Of note, miR-375 was identified in both analyses as the most significantly different miRNA, whose prevalence in circulation appeared to reflect better clinical outcome, i.e., pCR to NCT and absence of relapse. In addition to the clinical outcome, miRNAs associated with the biopathological characteristics of primary BC were also identified. A partial list (FDR < 0.05) of miRNAs significantly correlated with the status of ER, PR, HER2 and inflammatory BC is indicated in Table 2 (see Additional file 3: Table S3 for a full list of all miRNAs with *P *< 0.05). Interestingly, higher levels of circulating miR-375 were linked to negative ER/PR status, positive HER2 status, and inflammatory BC, whereas higher levels of circulating miR-122 were associated with HER2-negative and non-inflammatory tumors (Tables 2 and Additional file 3: S3).

**Table 1:** Circulating miRNAs associated with clinical outcome in stage II-III BC patients*

Metastatic Relapse in All patients in the Study Cohort				
	**Relapsed**	**Non-relapsed**	**Log fold diff. (relapsed vs. non-relapsed)**	
			
**Game name**	**(*N *= 11)**	**(*N *= 31)**		***P *value**
		
	**Ave. counts (normalized)**	**Ave. counts (normalized)**		

**hsa-miR-375**	2, 177	14, 316	-1.90	5.89E-05

**hsa-miR-122**	532, 501	168, 532	1.35	2.98E-03

**NCT Response in HER2^+ ^Patients**				

	**pCR**	**Non-pCR**	**Log2 fold diff (pCR vs. non-pCR)**	
		
**Game name**	***(N = 12)***	**(*N *= 11)**		**P value**
			
	**Ave. counts** **(normalized)**	**Ave. counts** **(normalized)**		

**hsa-miR-375**	32, 629	4, 014	3.02	1.10E-09

**hsa-miR-184**	245	31	2.99	2.59E-09

**hsa-miR-1299**	454	89	2.35	1.24E-06

**hsa-miR-196a**	989	239	2.05	1.79E-05

**hsa-miR-381**	384	1, 235	-1.69	2.48E-04

**hsa-miR-410**	129	364	-1.50	1.09E-03

**hsa-miR-1246**	19, 969	55, 915	-1.49	1.16E-03

**Table 2:** Circulating miRNAs associated with histopathological features of BC*

ER+ vs. ER-					
**Gene name**	**ER+ (*N = *21) Ave. counts**	**ER- (*N = *21) Ave. counts**	**Log^2 ^fold diff. (ER+ vs. ER-)**	***P *value**	**FDR**

**hsa-miR-375**	3, 189	18, 356	-2.23	6.28E-10	3.46E-07

**hsa-miR-429**	58	221	-1.94	7.48E-08	1.28E-05

**hsa-miR-196a**	184	694	-1.92	8.14E-08	1.28E-05

hsa-miR-141*	21	75	-1.82	5.99E-07	6.59E-05

**hsa-miR-376a**	200	65	1.63	4.72E-06	4.33E-04

**hsa-miR-370**	585	198	1.56	9.98E-06	8.44E-04

**hsa-miR-141**	442	1, 216	-1.46	3.34E-05	2.45E-03

**hsa-miR-200b**	150	400	-1.42	5.82E-05	3.37E-03

**hsa-miR-125a-5p**	2, 222	914	-1.28	2.55E-04	1.17E-02

**hsa-miR-205**	83	196	-1.24	4.43E-04	1.87E-02

**hsa-miR-200a**	218	482	-1.14	1.11E-03	4.08E-02

**hsa-miR-224**	870	402	1.12	1.39E-03	4.79E-02

**PR+ vs. PR-**					

Gene name	PR^+ ^(*N = *14) Ave. counts	PR- (*N = *28) Ave. counts	Log2 fold diff. (PR^+ ^vs. PR-)	*P *value	FDR

**hsa-miR-216a**	56	17	1.75	1.20E-06	1.56E-04

**hsa-miR-184**	28	115	-2.03	1.28E-06	1.56E-04

**hsa-miR-196a**	160	578	-1.85	5.80E-06	5.28E-04

**hsa-miR-370**	699	238	1.55	6.24E-06	5.28E-04

**hsa-miR-375**	4, 457	14, 477	-1.70	2.55E-05	1.76E-03

hsa-miR-376a*	225	86	1.40	5.27E-05	3.22E-03

**hsa-miR-133a**	84	32	1.36	8.72E-05	4.57E-03

**hsa-miR-654-5p**	154	64	1.25	2.88E-04	1.29E-02

**hsa-miR-125a-5p**	2, 550	1077	1.24	2.93E-04	1.29E-02

**hsa-miR-224**	1, 028	440	1.22	3.72E-04	1.52E-02

**hsa-miR-217**	120	52	1.22	4.97E-04	1.89E-02

**hsa-miR-429**	67	176	-1.38	5.72E-04	2.10E-02

**hsa-miR-494**	255	114	1.16	7.86E-04	2.62E-02

**hsa-miR-99b**	10, 464	4934	1.08	1.56E-03	4.40E-02

**HER2+ vs. HER2-**					

**Gene name**	HER2+ (*N *= 23) Ave. counts	HER2+ (*N *= 19) Ave. counts	Log2 fold diff. (HER2+. vs. HER-)	*P *value	FDR

**hsa-miR-885-3p**	32	160	-2.31	2.05E-10	1.13E-07

**hsa-miR-122**	98, 310	464, 256	-2.24	3.08E-10	1.13E-07

**hsa-miR-375**	17, 052	3, 977	2.10	1.72E-08	4.74E-06

**hsa-miR-184**	128	35	1.88	5.72E-07	1.05E-04

hsa-miR-1228*	65	166	-1.35	1.32E-04	7.62E-03

**hsa-miR-483-5p**	194	484	-1.32	1.60E-04	8.80E-03

**hsa-miR-429**	193	75	1.37	1.81E-04	9.48E-03

**hsa-miR-205**	189	80	1.23	7.03E-04	3.14E-02

**hsa-miR-217**	49	106	-1.12	1.41E-03	5.00E-02

**Inflammatory vs. Non-inflammatory**					

**Gene name**	Inflam. (*N *= 10) Ave. counts	Non-inflam. (*N *= 32) Ave. counts	Log2 fold diff. (Inflam. vs. Non inflam.)	*P *value	FDR

**hsa-miR-375**	35, 465	3, 535	3.33	3.76E-20	4.14E-17

**hsa-miR-184**	267	29	3.18	5.28E-18	2.90E-15

**hsa-miR-196a**	1217	195	2.64	3.62E-13	1.33E-10

**hsa-miR-429**	341	77	2.15	4.14E-09	5.69E-07

**hsa-miR-200a**	811	206	1.98	6.26E-08	7.65E-06

**hsa-miR-200c**	3, 714	1, 033	1.85	4.22E-07	4.65E-05

**hsa-miR-3065-5p**	86	29	1.56	3.11E-05	3.45E-04

**hsa-miR-141**	1, 571	597	1.40	1.44E-04	7.19E-03

**hsa-miR-203**	403	155	1.38	1.93E-04	8.85E-03

hsa-miR-200b*	89	36	1.33	3.90E-04	1.59E-02

**hsa-miR-1308**	5, 332	2, 358	1.18	1.35E-03	4.96E-02

*FDR < 0.05

### PCR validation of selected miRNAs

We next selected several miRNAs, including miR-375, miR-122, miR-184, miR-196a, miR-1, miR-410, miR-432, and miR-16, for qRT-PCR-based validation using the same total RNA extracts used for deep sequencing (32 out of the 42 sequenced samples had sufficient amounts left for PCR assays). The sequencing-determined abundance of these miRNAs ranged from very high (e.g., miR-122, ranged from 4, 683-1, 094, 999 counts with an average of 16, 456) to very low (e.g., miR-184, ranged from 6-1, 097 counts with an average of 61, and miR-410, ranged from 1-1, 620 counts with an average of 220). For PCR analyses, levels of miR-16, which were consistent among all samples (Additional file 2: Table S2), and reportedly used as the internal control for circulating miRNAs in previous PCR-based studies [22,24], were used as the reference for data normalization. Our results indicated that gene-specific PCR could detect miRNA with as few as 20 counts in a sample (data not shown), and the low-abundant miR-184 and miR-410 could be detected from ~90% of all tested samples (29 out of 32). Pairwise Pearson correlation was calculated to determine the consistency of the miRNA levels determined by deep sequencing (normalized log2 counts and PCR-determined levels relative to miR-16) in each sample. For all tested miRNAs, significant (*P *< 0.05) correlations were observed between the two methods (Additional file 1: Figure S2).

**Figure 2:**
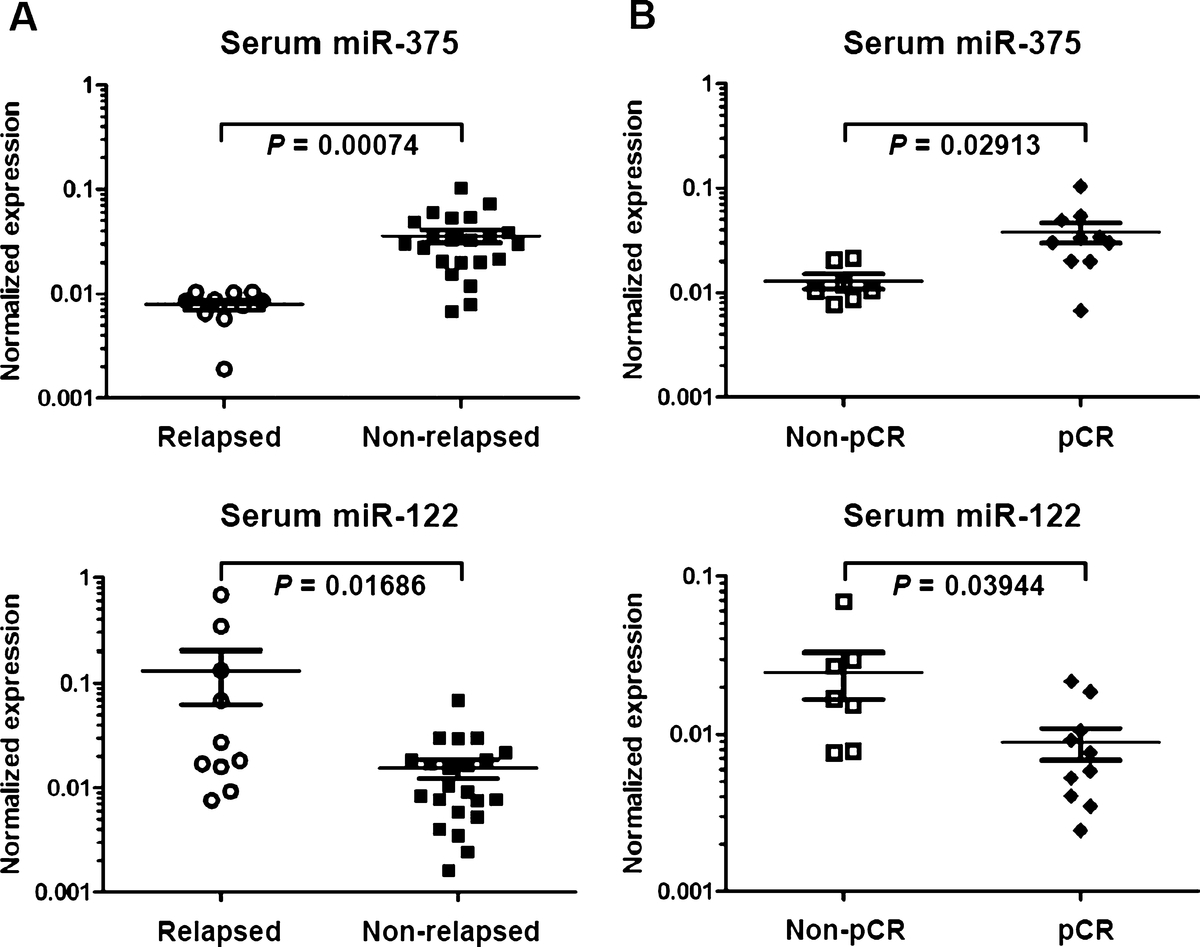
**PCR-determined levels of miR-375 and miR-122 are associated with clinical outcome**. Levels of miR-375 and miR-122 were measured by PCR and normalized to the level of miR-16 in each sample. Comparisons were carried out between the two groups of relapsed vs. non-relapsed (**A**) or pCR vs. non-pCR (**B**). For each group, mean and the standard error of the mean (SEM) are indicated. *T*-tests were performed between the two groups and *P *values are indicated.

We further focused on miR-375 and miR-122, two miRNAs with the most significant fold differences in the associations with metastatic relapse and NCT response (Table 1). Consistent with the deep sequencing results, lower levels of miR-375 and higher levels of miR-122 detected by PCR both significantly correlated with disease relapse in all patients and with resistance to NCT (non-pCR) in HER2^+ ^patients (Figures 2 and Additional file 1: Figure S3). These data indicated the feasibility of cost-efficient PCR assays of these two genes to potentially predict clinical outcome of locally advanced BCs. Levels of other miRNAs examined by PCR did not show significant differences in the comparisons for disease relapse and NCT response (data not shown).

### Prediction of relapse by circulating miR-375 and miR-122

We next evaluated the sensitivity and specificity of the two circulating miRNA markers we identified, i.e., miR-375 and miR-122, in predicting metastatic relapse in our NCT study cohort using leave-one-out cross validation (See method section for details). Results indicated that both circulating miR-375 and miR-122 could predict metastasis with relatively high sensitivity and specificity, and the miR-375/miR-122 two-gene signature demonstrated the best predicting performance, with a sensitivity of 80% and specificity of 100% (Table 3).

**Table 3:** Performance analysis among training and testing cohorts

Training Cohort (*N *= 32)					
**Predictor**		**Predicted Relapse**	**Predicted Non-relapse**	**Sensitivity**	**Specificity**

**Circulating miR-375**	Observed relapse	9	1	9/10 (90%)	20/22 (91%)

	Observed non-relapse	2	20		
**Circulating miR-122**	Observed relapse	4	6	4/10 (40%)	11/22 (50%)

	Observed non-relapse	11	11		

**375/122 two-gene signature**	Observed relapse	8	2	8/10 (80%)	22/22 (100%)

	Observed non-relapse	0	22		

**Training Cohort (*N *= 26)**					

**Predictor**		**Predicted Relapse**	**Predicted Non-relapse**	**Sensitivity**	**Specificity**

**Circulating miR-375**	Observed relapse	0	8	0/8 (0%)	11/18 (61%)

	Observed non-relapse	7	11		

**Circulating miR-122**	Observed relapse	7	1	7/8 (88%)	14/18 (78%)

	Observed non-relapse	4	14		

**375/122 two-gene signature**	Observed relapse	2	6	2/8 (25%)	17/18 (94%)

	Observed non-relapse	1	17		

### Circulating miR-122 predicts metastasis in an independent cohort of early-stage BCs

**Figure 3:**
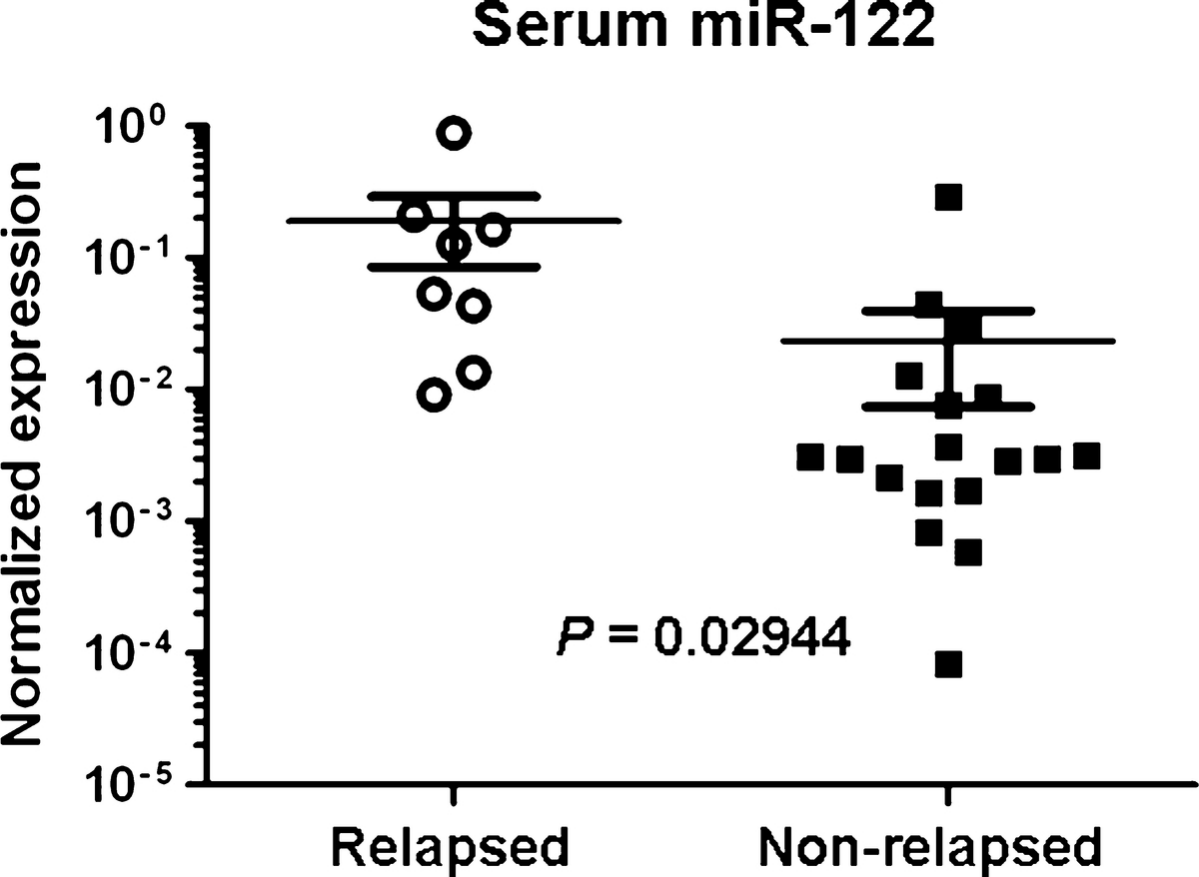
**Circulating miR-122 predicts metastatic relapse in an independent BC cohort**. Levels of serum miR-122 in an independent validation cohort of 26 stage II-III BC patients were measured by PCR and normalized to the level of miR-16 in each sample.

We further assembled an independent validation cohort of 26 stage II-III BC patients, including 8 patients with metastatic recurrence within 2 years after initial diagnosis, as well as appropriate controls with matched clinical parameters but without disease recurrence (Additional file 1: Table S1). Serum RNA was isolated and subjected to qRT-PCR to detect levels of miR-375, miR-122 and miR-16. Upon normalization to miR-16, circulating miR-122 levels were significantly higher in the group with relapse (*P *= 0.0294), and could predict metastasis at a sensitivity of 88% and specificity of 78% in this cohort (Figure 3 and Table 3). Levels of miR-375, however, were not significantly different between the relapsed and non-relapsed groups (Table 3 and data not shown), possibly due to the fact that patients in the validation cohort were generally lower stage at diagnosis, with more hormone receptor positive disease, and more frequently overexpressed HER2 (Additional file 1: Table S1). In addition, they received diverse therapies seen in a general oncology practice. Because the status of ER/PR and HER2 in primary BCs have been historically linked to clinical outcomes [30,31], we also computed the sensitivity and specificity of each histopathological parameter in association with development of metastatic relapse in the testing cohort. Results indicated that, in comparison to the histopathological parameters, circulating miR-122 served as a better predictor of metastasis, regardless of the heterogeneity of this cohort (Odds Ratio 24.5; *P *< 0.01; Additional file 1: Table S4).

## Discussion

Using a comprehensive *de novo *sequencing approach, we identified sets of circulating miRNAs that were associated with various clinicopathological parameters and clinical outcome in stage II-III BC patients. Previous studies have linked higher circulating levels of miR-10b and miR-21 to negative ER status [22], and higher circulating levels of miR-155 to positive PR status [25]. None of these miRNAs exhibited correlations with ER/PR status in our study, possibly due to the differences of the size, composition of study cohorts, and/or treatment regimens. We observed that two miRNA clusters, miR-200b-200a-429 and miR-200c-141, were significantly associated with negative ER status and inflammatory BC (Tables 2 and Additional file 3: S3). In addition, we found that several miRNAs, including miR-375, miR-429, miR-196a, miR-370, miR-125a-5p, and miR-224, simultaneously correlated with both ER and PR status in the same direction (Table 2). Among these miRNAs, miR-375 and miR-429 also correlated with HER2 status but in the opposite direction as compared to their correlations with ER/PR status (Table 2). Expression of these miRNAs in primary BCs and their functional links with ER/PR/HER2 merit additional investigation, and may further elucidate the pathogenic mechanisms of these long-known receptors in BC.

We also identified a two-gene signature consisting of miR-375 and miR-122 with the capacity to predict disease relapse in our study cohort of stage II-III BC patients who received identical NCT regimens. In a heterogeneous validation set derived from an observational cohort, circulating miR-122, but not miR-375, remained as a predictor of metastasis (Figure 3). Interestingly, both miR-375 and miR-122 correlated with HER2 status (Table 2), with higher levels of miR-375 and lower levels of miR-122 associating with positive HER2 status (Table 2), pCR to NCT (Figure 2B), and absence of relapse (Figure 2A). Consistent with previous observations that HER2^+ ^BCs have higher rates of pCR to NCT [30], these results raise interesting questions to be addressed in future studies. For example, the origin of circulating miRNA is still unclear. It has been proposed that tumor-associated miRNAs can be released into the bloodstream when tumor cells are dying and being lysed [22,32], or through active secretion of miRNA-loaded exosomes by tumor cells [20]. Furthermore, the cellular source of circulating miR-375 and miR-122 remains unknown; their presence may reflect expression in the primary tumor or in other cell types, such as immune cells.

MiR-122, the circulating miRNA that consistently predicted metastasis in both our study cohort and validation cohort, is the most frequent miRNA isolated in the liver and is involved in the regulation of lipid metabolism [33]. Downregulation of miR-122 has been reported in hepatocarcinoma (HCC) [34]. In contrast, higher levels of miR-122 in circulation correlate with HCC [35] and liver injury [36]. Expression of miR-122 has also been reported in primary fibroblasts, where the miRNA is involved in p53 mRNA polyadenylation/translation by targeting the cytoplasmic polyadenylation element binding protein (CPEB) [37]. Expression and function of miR-122 have not yet been reported in BC. Our results here, however, strongly suggest a role of miR-122 in BC progression, an area of study currently under investigation in our laboratory.

Roth et al. report that circulating levels of miR-10b, miR-34a and miR-155 correlate with the presence of overt metastases in BC patients [24]. These miRNAs, however, did not significantly correlate with metastatic relapse in our retrospective study in stage II-III patients. It is possible that the change of circulating miR-10b, miR-34a and miR-155 levels occurs after cancer dissemination, whereas levels of circulating miR-122 and miR-375, as identified herein, start to change and reflect metastatic potential at an earlier stage of disease. Validation using a larger patient cohort will be necessary in future studies. Findings will allow us to further refine a circulating miRNA signature that can predict metastasis and guide individualized adjuvant and neoadjuvant therapy to minimize risk of systemic relapse.

## Conclusions

Using a comprehensive *de novo *sequencing approach, we identified sets of circulating miRNAs that were associated with various clinicopathological parameters and clinical outcome in stage II-III BC patients. We also identified a two-gene signature consisting of miR-375 and miR-122 with the capacity to predict disease relapse in our study cohort of stage II-III BC patients who received identical NCT regimens. In a heterogeneous validation set derived from an observational cohort, circulating miR-122, but not miR-375, remained as a predictor of metastasis. These results may allow optimized chemotherapy treatments and preventive anti-metastasis interventions in future clinical applications.

## Competing interests

The authors declare that they have no competing interests.

## Authors' contributions

Conception and design: SEW, GS (Somlo), YY (Yen), JJR, MRP, XR. Provision of study materials or patients: GS (Somlo), MRP, YY (Yen). Collection and assembly of data: XW, YY (Yu), WZ, AC, HG, JW, GS (Sun), YW, RS, KR. Data analysis and interpretation: XW, AXL, YCY, PF, SL, KTA, XR. Manuscript writing and approval: All authors

## Supplementary Material

Additional file 1 **Figure S1Composition of small RNA sequences in a representative serum sample**. Total sequence reads obtained from deep sequencing were aligned to human genome database NCBI36/hg18. The percentage of each class of small RNAs was indicated in the pie chart. scRNA: small cytoplasmic RNA; snRNA: small nuclear RNA; snoRNA: small nucleolar RNA; mt-tRNA: mitochondrial tRNA. Click here for file

Additional file 2 **Additional file 2: Table S2**. Normalized counts of all miRNAs in the circulation. Click here for file

Additional file 3 **Additional file 2: Table S3**. Full lists of miRNAs associated with biopathological parameters. Click here for file
