# Supplementary material for: De novo sequencing of circulating miRNAs identifies novel markers predicting clinical outcome of locally advanced breast cancer
Source: J Transl Med. 2012 Mar 8;10:42. doi: 10.1186/1479-5876-10-42 (PMC3342150; doi:10.1186/1479-5876-10-42)
Supplement: Additional file 1 — Figure S1Composition of small RNA sequences in a representative serum sample. Total sequence reads obtained from deep sequencing were aligned to human genome database NCBI36/hg18. The percentage of each class of small RNAs was indicated in the pie chart. scRNA: small cytoplasmic RNA; snRNA: small nuclear RNA; snoRNA: small nucleolar RNA; mt-tRNA: mitochondrial tRNA. [file 1479-5876-10-42-S1.PDF]

Supplementary Material

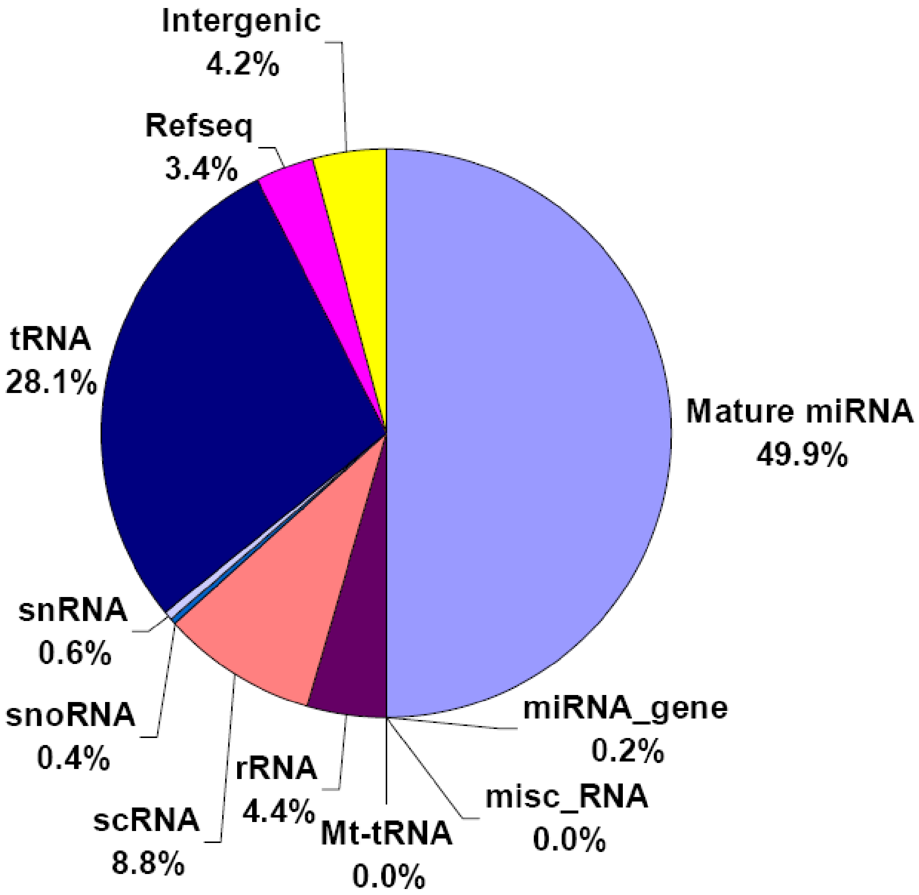

Figure S1. Composition of small RNA sequences in a representative serum sample. Total sequence reads obtained from deep sequencing were aligned to human genome database NCBI36/hg18. The percentage of each class of small RNAs was indicated in the pie chart. scRNA: small cytoplasmic RNA; snRNA: small nuclear RNA; snoRNA: small nucleolar RNA; mt-tRNA: mitochondrial tRNA.

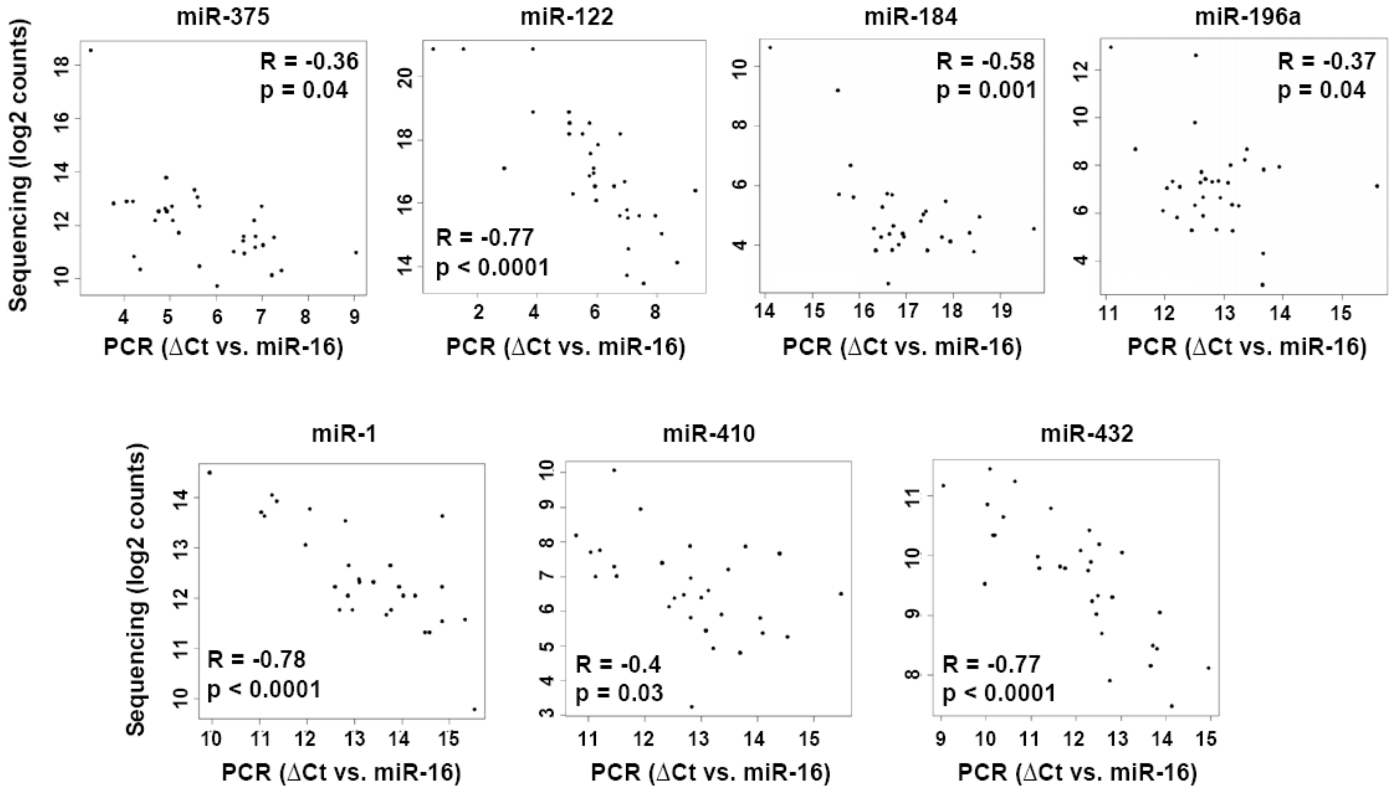

Figure S2. Correlation between miRNA levels determined by sequencing and PCR. Pairwise Pearson correlation was calculated to determine the consistency of the miRNA levels determined by deep sequencing (normalized counts in log2) and PCR (ΔCt vs. miR-16) in each sample. Pearson correlation coefficients (R) and *P* values are indicated for each miRNA.

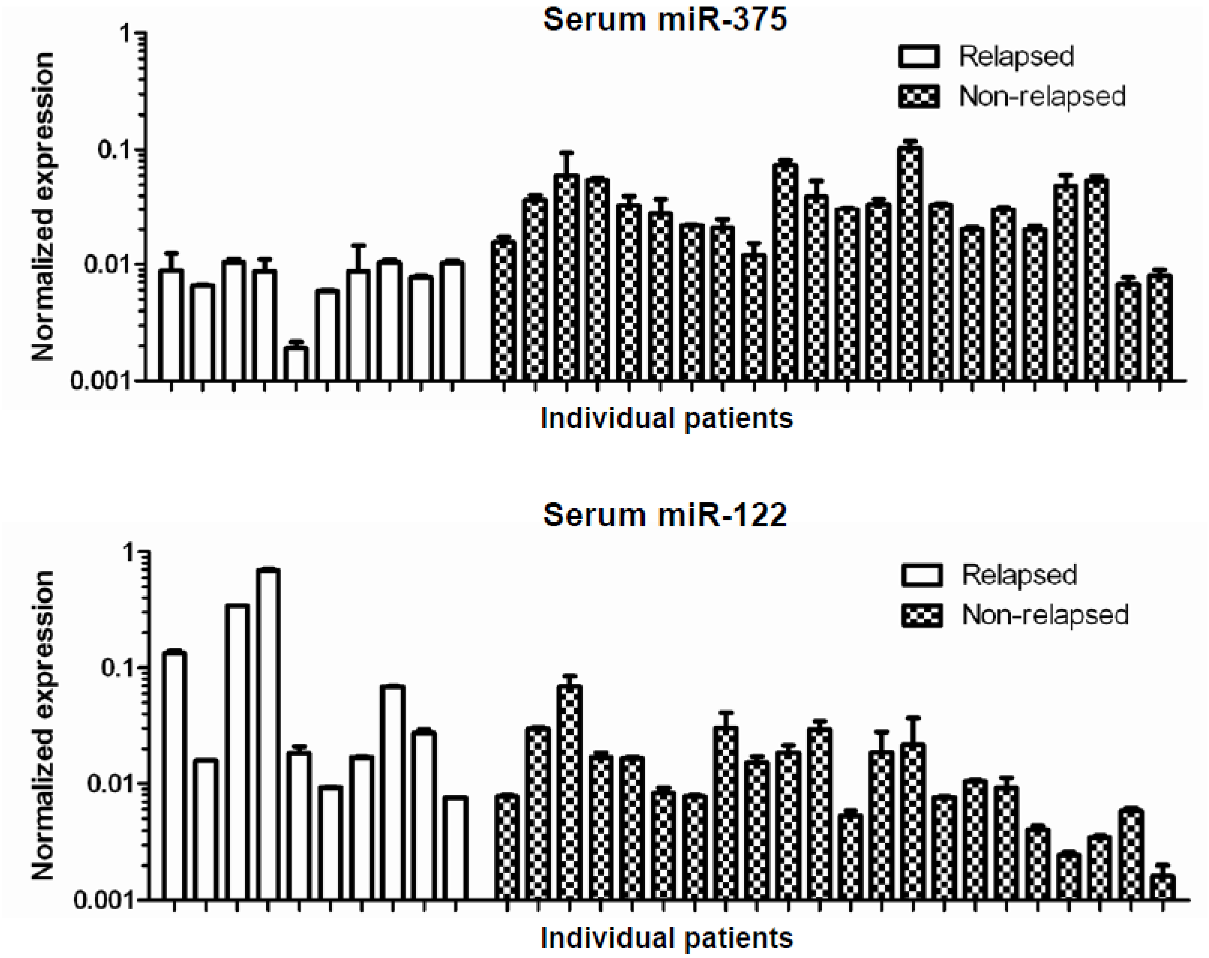

Figure S3. Relative levels of miR-375 and miR-122 in individual serum samples in the study cohort. PCR-determined levels of miR-375 and miR-122 were normalized to miR-16 in each sample. Each column indicates the average miRNA level from triplicate PCR reactions. Standard deviations (SD) are indicated as error bars.

Table S1. Clinical characteristics of patients in the training and testing cohorts

		Training cohort *N* (%)	Testing cohort *N* (%)
**Patient**	**(*N*)**	42	26
**Age**	**(Mean ± SD)**	53.2 ± 10.1	52.7 ± 9.1
**ER**	**Positive**	21 (50)	22 (85)
	**Negative**	21 (50)	4 (15)
**PR**	**Positive**	14 (33)	15 (58)
	**Negative**	28 (67)	11 (42)
**HER2**	**Positive**	23 (55)	20 (77)
	**Negative**	19 (45)	6 (23)
**Stage**	**II**	15 (36)	16 (62)
	**III**	26 (62)	10 (38)
	**IV**	1 (2)	0 (0)

Table S4. Univariate analysis of predictors' association with progression in test set (*N* = 26)

Predictor		Relapse *N* (%)	Non-relapse *N* (%)	OR	95% CI	*P* value
**Circulating miR-375**	Predicted relapse	0 (0)	7 (38.9)	0	0 – Inf	0.99
	Predicted non-relapse	8 (100)	11 (61.1)			
**Circulating miR-122**	Predicted relapse	7 (87.5)	4 (22.2)	24.5	2.3 – 262.5	0.008
	Predicted non-relapse	1 (12.5)	14 (77.8)			
**375/122 two-gene signature**	Predicted relapse	2 (33.3)	1 (5.6)	5.7	0.4 – 74.4	0.19
	Predicted non-relapse	6 (66.7)	17 (94.4)			
**ER**	Positive	6 (75)	16 (88.9)	0.4	0.04 – 3.3	0.38
	Negative	2 (25)	2 (11.1)			
**PR**	Positive	3 (37.5)	12 (66.7)	0.3	0.05 – 1.7	0.17
	Negative	5 (62.5)	6 (33.3)			
**HER2**	Positive	3 (37.5)	3 (16.7)	3.0	0.5 – 19.9	0.26
	Negative	5 (62.5)	15 (83.3)			
**Stage**	II	5 (62.5)	11 (61.1)	1.1	0.2 – 5.9	0.95
	III	3 (37.5)	7 (38.9)			
